# Sol–gel silica coatings for enhanced silicon emission: microstructural origins and optical implications

**DOI:** 10.1039/d5na00472a

**Published:** 2025-10-22

**Authors:** Inas Taha, Sufian Abedrabbo, I. A. Qattan, El Mostafa Benchafia, Mohammad Khaled Shakfa, Dalaver H. Anjum

**Affiliations:** a Department of Physics, College of Engineering and Physical Sciences, Khalifa University of Science and Technology PO Box 127788 Abu Dhabi United Arab Emirates dalaver.anjum@ku.ac.ae sufian.abedrabbo@ku.ac.ae

## Abstract

Silicon is an attractive platform for optoelectronic integration, but its indirect bandgap makes it a weak light emitter. Here, we demonstrate that sol–gel-derived silica (SiO_2_) coatings, when thermally annealed, can significantly boost silicon bandgap photoluminescence (PL). Samples annealed at 900 °C exhibit a more than fourfold increase in emission intensity near 1160 nm compared to as-deposited films. High-resolution Transmission Electron Microscopy (TEM), combined with geometric phase analysis (GPA), revealed that as-deposited samples exhibit relatively uniform interfacial strain of less than ±0.1%, whereas annealing at 900 °C introduces local strain fluctuations of up to ±2.0%. These nanoscale strain variations correlate directly with the observed PL enhancement. Detailed analysis using high-resolution TEM (HRTEM) and scanning transmission electron microscopy with electron energy-loss spectroscopy (STEM-EELS) reveals the reduction of annealing-induced defects in Si and the densification of the silica layer. These modifications alter the electronic states at the interface, as reflected in changes in the joint density of states, thereby enabling more efficient radiative recombination. Finite difference time domain (FDTD) simulations suggest that the enhanced PL signal near 1160 nm is partly attributable to annealing-induced morphological changes in silica. Together, these findings demonstrate that sol–gel-derived SiO_2_/Si stacks provide a simple and low-cost route to enhance silicon emission through strain engineering, offering strong potential for integration into CMOS-compatible photonic systems and the development of future on-chip light sources and optical interconnects.

## Abstract 2

Silicon is an attractive platform for optoelectronic integration, but its indirect bandgap makes it a weak light emitter.

## Graphical abstract

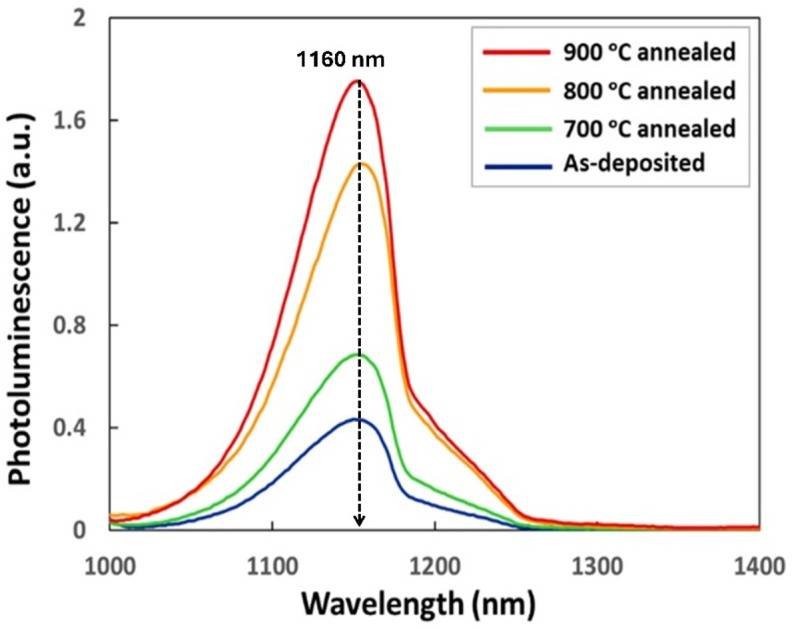

## 1 Introduction

Silicon (Si) is one of the most important semiconductors for various electronic applications. It is reasonably low-cost and has very stable dielectric properties. It also has a bandgap value of 1.1 eV, making it an excellent detector in many analytical techniques. Moreover, Si is an extensively used material in integrated circuits (ICs) for electronic applications. The future trend of increased device speed and functionality stipulates less all-electronics and the need for on-chip and chip-to-chip optical data transmission. Therefore, the IC industry actively explores the photonic properties of Si and is working on the development of Si-photonic devices. This establishes the need for an efficient Si emitter material or stacks that monolithically blend with other IC components.^1,2^

The challenge lies in the fact that Si is ordinarily a weak light emitter.^3,4^ This is attributed to its nature as an indirect bandgap semiconductor, where non-radiative recombination paths such as Auger recombination are dominant over bimolecular radiative recombination of free-carriers.^5^ In 1952, Kingsbury and Ohl discovered the phonon-mediated light emission from bulk Si, where limitations of the indirect band gap result in a three-particle inverse of the photoelectric effect.^6^ The bimolecular radiative recombination coefficient, *β* = 3 × 10^15^ cm^−15^ s^−1^ determines the desirable radiative recombination process that competes with faster undesirable non-radiative recombination processes. The quantum efficiency *η* ≈ *βn*(*τ*_NR_^−1^ + *βn* + *γn*^2^)^−1^, where *τ*_NR_^−1^ is the non-radiative decay rate, *n* is the charge carrier density, and *γ* is the Auger coefficient, can reach a theoretical value of 1% under reasonably high injection of carriers, indicating that harvesting light from Si is not impossible.^7,8^

Significant research efforts have been undertaken to enhance the light emission from silicon, aiming to identify an efficient light-emitting candidate. One strategy involves engineering strain fields to induce radiative recombination among carriers in Si quantum emitters, as demonstrated by Restori *et al.*^9^ Computational efforts to understand the role of strain^10^ have also been made. Collecting light emitted from nano-crystals of Si has also attracted interest, as manifested by the recent work of Ma *et al.*^11^ More interestingly, Si was strained by alloying with Ge synthesizing Si/Ge alloys.^12^ More recently, strain was utilized to induce variations in the emission of Si-quantum-dots.^13^ Impurity centers, such as carbon-based G-centers, have also been investigated because they emit at sub-bandgap energies and therefore experience minimal reabsorption in the Si host.^14^ Interestingly, telecom-interfaced solid-state spins at the O-band are another interesting range of wavelengths where Si becomes a window. Johnston *et al.*^15^ explored the novel atomic defect T-centers, which are defined as a carbon–carbon pair with a hydrogen atom bonded to one of the carbon atoms. At the same time, the other C-atom possesses an unpaired electron. Related to impurity centers, the C-band remains the preferred choice for amplification, specifically using erbium. Notable advances include the demonstration of erbium–silicon waveguides in Cz-Si and float-zone Si by Gritsch *et al.*,^16^ and the observation of long electron and optical spin coherence times in isotopically purified Si by Berkman *et al.*^17^ In parallel, silica on silicon has long been studied due to its role as the gate oxide in MOS devices and its widespread use as an interlayer dielectric in integrated circuits. Mastalieva *et al.* demonstrated the ability to generate 2nd harmonic broadband photoluminescence (PL) in mesoporous Si/SiO_2_ nanoparticles.^18^ At this juncture, one cannot proceed without relating the recent noteworthy effort performed on Er-impurity centers in silica on Si, particularly SiO_2_ deposited using sol–gel techniques. In this case, the device is Si-related, but the impurity center is in the silica dielectric. A comprehensive overview of such sol–gel-derived dielectric materials was provided by Raghini *et al.*^19^

To this end, Abedrabbo *et al.* have been attempting to circumvent the effect of the indirect bandgap of Si to increase its light emission, where they have successfully utilized ion-beam mixing techniques forced by an energetic Ar^+^ beam to introduce Si–Ge structures for photovoltaic applications initially.^8^ Following that strategy, they leveraged the same method to achieve GeO_2_ nanostructures in Si as an optoelectronic candidate, followed by the incorporation of rare-earth impurity centers to achieve C-band 4f emission at 1.5 μm.^20–22^ Starting from 2009, Abedrabbo's group followed an unorthodox technique of introducing random strains into the Si surface by coating it with sol–gel-based erbium-doped SiO_2_, which is catalyzed by various acids. This approach unexpectedly enhanced the bandgap-related emission intensity by two orders of magnitude.^23^ A multi-phonon three-particle model modeled the emission spectra, and comparative studies with other reported room-temperature Si PL have all proved that the recombination of electron–hole carriers is indeed originating from the bandgap.^24–26^ The sol–gel-based silica films were extensively studied, including the effect of thickness, processing parameters like annealing temperature, and even Er as a dopant on films, including polarizability effects and its possible role in Si bandgap enhanced emission.^27–30^ These studies are crucial to understanding the role of spin-coated silica, whether doped or undoped, in Si bandgap emission. However, despite an extensive literature review, there is a lack of structural exploration of sol–gel-based silica on Si. This limits our understanding of how processing methodologies influence the interfacial states and structural properties of films, particularly in cases of bandgap-enhanced emission. This gap hampers the development of next-generation waveguide devices. Thus, the significance of this study is underscored, which could provide the first comprehensive analysis of these samples.

Recent progress in sol–gel-derived nano/mesoparticles and ceramic nanocomposites further contextualizes our study. Sol–gel routes have been used to engineer mesoporous SiO_2_ nanopowders for ceramic filtration and nanocomposite applications, where pore architecture and particle size directly impact device-level performance (*e.g.*, nanomullite/SiC ceramic filters).^31^ Beyond oxides, sol–gel precursors combined with carbothermal reduction have enabled the synthesis of carbide nanopowders such as TiC, with pH and temperature shown to critically govern phase formation and crystallite size, highlighting processing-structure–property linkages comparable to our SiO_2_/Si stacks.^32^ In cementitious/ceramic matrices, sol–gel-derived fillers are increasingly incorporated into geopolymeric binders; for example, metakaolin-red-mud/CNT geopolymers demonstrate how nanoscale additives bridge cracks and refine porosity to enhance mechanical response.^33^ At the porous-ceramic frontier, dual-network aerogel architectures with multiscale pores provide a clear example of how controlled micro/meso-porosity tunes electromagnetic absorption and transport, which parallels our observation that morphology (void-rich *vs.* densified silica films) modulates the optical response.^34^ Finally, in the broader context of construction materials, a study on CaO/MgO-driven phase formation in ceramic bodies has revealed how chemistry and thermal history determine phase evolution, strain, and durability concepts, which are mirrored in our annealing-driven densification and variations in JDOS.^35^ Collectively, these studies reinforce two points central to our results: (i) sol–gel processing affords powerful levers (pH, temperature, and precursor chemistry) to sculpt nano/mesostructured and interfaces; (ii) morphological/electronic changes (densification, defect evolution, and porosity) drive functional gains – from mechanics and EM response to the optical emission enhancement we observe after 900 °C annealing.

In this paper, we present a novel approach that utilizes Transmission Electron Microscopy (TEM) characterization to thoroughly analyze sol–gel-based SiO_2_/Si structures, shedding light on the microstructural properties at the interface that enable bandgap emission enhancement and the development of future Si-based optoelectronic devices. Additionally, finite-difference time-domain (FDTD) simulations were conducted to establish the optical response of the SiO_2_ layer in samples.

## 2 Experimental methods

The silica films were synthesized using a typical sol–gel procedure with an acid catalyst to speed the gelation time. The process involved using the Si precursor (Sigma-Aldrich – tetraethyl orthosilicate (TEOS), 99.99%) and deionized water, with Honeywell ethanol (EtOH, 99.8%) as the solvent and phosphoric acid (Sigma-Aldrich H_3_PO_4_, ≥85). The TEOS precursor was first mixed with ethanol, followed by the addition of water. The molar ratio for the constituents was: TEOS : water : ethanol : acid = 1 : 4 : 9.59 : 0.05. The chosen ratio ensures complete hydrolysis and condensation. Phosphoric acid is known to promote low-porosity films with accelerated gelation while preventing uncontrolled precipitation, in line with reported sol–gel silica chemistry. The solution was stirred at 300 rpm for 3 hours at room temperature, followed by an additional 15 minutes at 75 °C after the addition of acid to promote further hydrolysis and condensation. The processed sol was then filtered and spin-coated onto (100) p-type 4′′ Cz-Si wafers (5–10 Ω cm resistivity) at 1000 rpm for 30 seconds, using an EZ-8 vacuum spin coater from Schwan Technologies, yielding films with a thickness of approximately 300–500 nm. The coated wafer was dried at 120 °C for 1 hour in a vacuum oven to remove residual solvents.^29,30^ Following this step, the wafer was scribed into several small substrates, annealed at various temperatures ranging from 500 to 900 °C, with one substrate left as-deposited (baked at 120 °C) as a reference sample. An MTI compact 5′′ split vacuum furnace was utilized for the thermal treatment while maintaining a moderate vacuum level of 10^−2^ Torr. In this study, only one as-deposited and one annealed at 900 °C sample, representing the extreme opposites, were used to perform extensive microstructural analysis. For the room-temperature PL studies, a Fluorolog Quanta Master (FL-QM) spectrofluorometer, supplied by Horiba Instruments, was used with a steady-state InGaAs detector and a 980 nm laser diode as the excitation source.

For TEM investigations on the stacks, thin slices of the SiO_2_/Si lamella-based structure were prepared using a dual-beam FIB tool. The TEM analysis used a model Titan 80–300 ST. The microscope was operated at an accelerating voltage of 300 kV. The Bright-field TEM (BF-TEM) imaging technique was used to determine stack integrity, morphology, and the thickness of each layer. High-resolution TEM (HRTEM) was applied to determine the stack structure at atomic resolution. Moreover, the analysis of HRTEM images was performed to determine the defect density in the substrates of both as-deposited and annealed samples. The strain field distributions along (*i.e.*, in the 〈1̄10〉 direction) and perpendicular (*i.e.*, in the 〈001〉 direction) were obtained by applying geometric phase analysis (GPA) to HRTEM images of SiO_2_/Si interfaces. Specifically, spatial frequencies, represented by (004) and (2̄20) lattice planes of the Si substrate that appear at 0.136 nm and 0.192 nm values in the fast-Fourier transform (FFT) of HRTEM images were utilized to generate strain maps in 〈001〉 and 〈1̄10〉 directions, respectively. Dark-field scanning TEM (DF-STEM) was combined with electron energy loss spectroscopy (EELS) to acquire STEM-EELS spectrum imaging (SI) datasets to determine the elemental distribution of elements present in the stacks and for probing the joint density of states (JDOS) of SiO_2_ layers. Furthermore, the application of Kramers–Kronig analysis (KKA) to STEM-EELS SI datasets allowed the investigation of the dielectric properties of the SiO_2_ layers.^36,37^ Similarly, the dielectric properties and JDOS of the silicon substrate were investigated through high-energy-resolution EELS analysis using another electron microscope, ThemisZ, which was equipped with an electron beam energy monochromator and a Continuum 1069HR energy filter in counting mode.

To investigate the effect of annealing temperature on the optical properties of the SiO_2_ layer, FDTD simulations were performed using the Lumerical 3D package. A plane wave source in the wavelength range of about 400–1300 nm was incident normally on the SiO_2_/Si interface, allowing for the determination of transmittance. Light reflectance plots were generated. The SiO_2_ layer was integrated into the Si substrate under simulation conditions, and a plane wave source was employed, pointing downward. Power monitors were strategically placed above the source and after the SiO_2_ layer to capture the reflected and transmitted waves, respectively. The optical response of the sol–gel derived SiO_2_ layers was simulated using the finite-difference time-domain (FDTD) method implemented in the Lumerical 3D package. Specifically, a plane wave source spanning 400–1300 nm was incident normally on the SiO_2_/Si interface to evaluate the transmittance and reflectance spectra. The SiO_2_ layer, approximately 500 nm thick, was modeled on a crystalline Si substrate consistent with experimental deposition conditions. Perfectly matched layer (PML) boundary conditions were applied along the propagation direction to eliminate artificial reflections, while periodic boundary conditions were imposed in the lateral directions. Power monitors placed above and below the SiO_2_ layer recorded the reflected and transmitted fields, respectively. The optical constants (refractive index *n* and extinction coefficient *k*) for Si and SiO_2_ were obtained from experimental ellipsometry data, and the mesh size was refined to ensure numerical accuracy and convergence.

## 3 Results and discussion

**Fig. 1:**
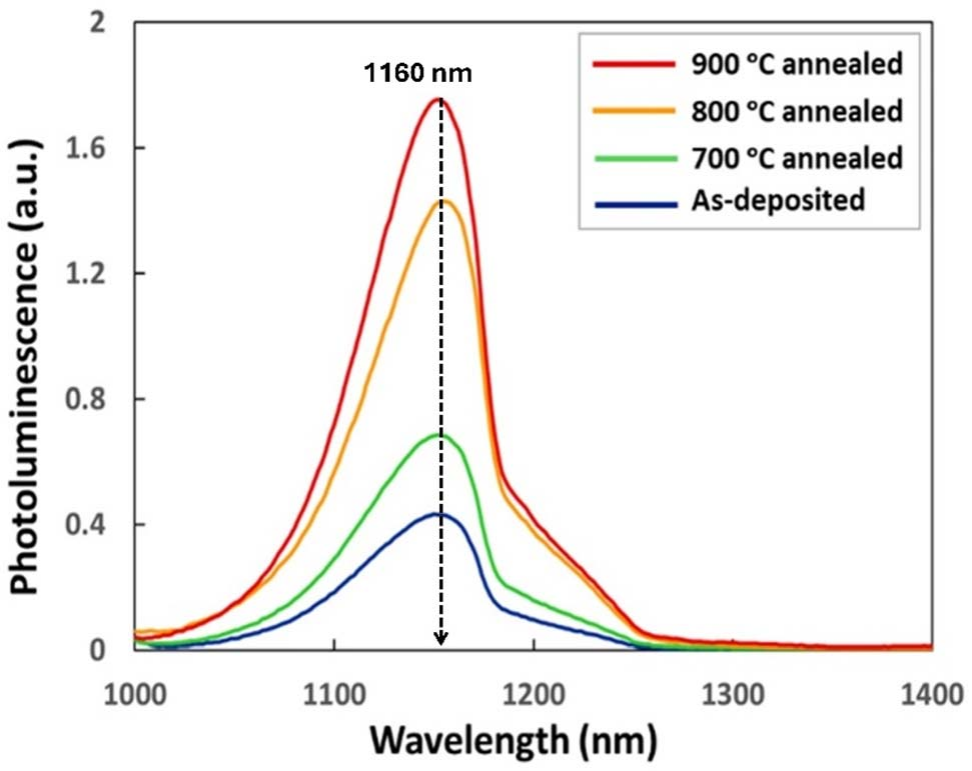
PL spectra of as-deposited, 700 °C, 800 °C, and 900 °C annealed samples. The presented data demonstrate an over four-fold increase in PL intensity of the 900 °C annealed samples, and its annotated peak position confirms that all samples emit light at the same wavelength of 1160 ± 10 nm.

The PL spectra acquired at room temperature for both the as-deposited samples and those annealed at temperatures of 700 °C, 800 °C, and 900 °C are presented in Fig. 1. Given that the samples annealed at 900 °C showed the most significant enhancement in the PL spectra, we focused solely on these samples, along with the as-deposited samples. It is observed that there is no visible shift in the highest intensity of the overlaid spectra for both the as-deposited and 900 °C samples, with the dominant peak remaining around 1160 nm. The same dominant peak position was observed in sol–gel-based silica coatings doped with Er, although processed differently. This indicates that sol–gel-based silica coatings (both doped/undoped) perturb the Si bandgap in a unique way that enhances radiative bandgap recombination while narrowing the gap by approximately 0.012 eV.^22,23^ The 900 °C annealed samples exhibit a four-fold increase in PL intensity compared to the as-deposited sample.

Overall, the emission intensities exhibited an upward trend with a distinctive peak, possibly indicating that the fundamental properties of the stacks may play a minor role in enhancing the PL intensity. The sol–gel process, while attractive for its simplicity and low-cost fabrication of oxide films, is inherently prone to thickness non-uniformity, particularly when applied to large-area silicon wafers. During spin coating or dip coating, variations in solvent evaporation rates, viscosity gradients, and capillary forces across the wafer surface can lead to uneven film deposition. Such non-uniform coatings can significantly influence the optical, mechanical, and electronic properties of the deposited oxide layer, making careful optimization of sol–gel parameters and post-deposition treatments essential for achieving reliable and reproducible films.

**Fig. 2:**
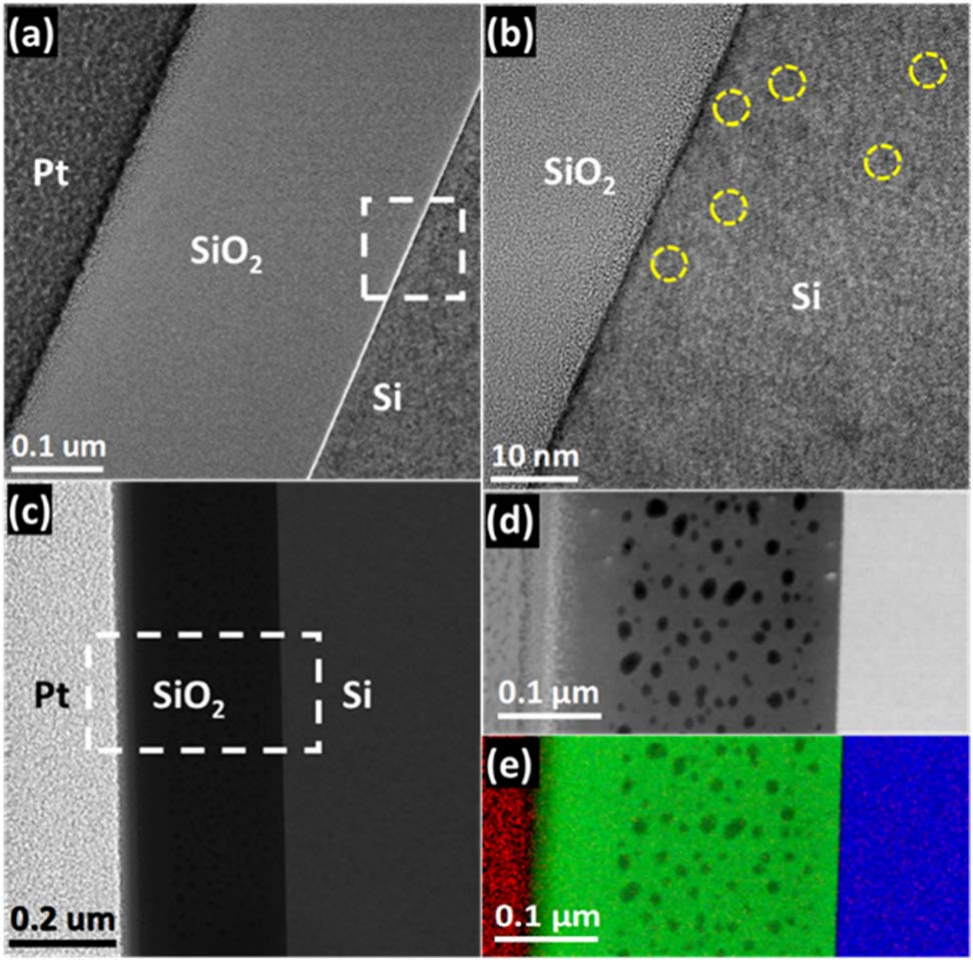
TEM analysis for the as-deposited sample: (a) cross-sectional low-resolution image revealing SiO_2_ thickness around 320 nm and (b) HRTEM image showing line defects in the Si region. The presence of point defects is marked by adding circular shape enclosed regions, (c) DF-STEM image exhibiting the presence of voids in the SiO_2_ layer, (d) DF-STEM image of the area enclosed by a rectangular white-color box in (c) that was selected for the acquisition of STEM-EELS SI datasets, and (e) RGB composite generated with Pt (red), O (green) and Si (blue) elemental maps of the area represented by the DF-STEM image in (d). The presence of voids in the SiO_2_ is also visible as dark regions.

To further understand the optical characteristics of the as-deposited and 900 °C annealed samples, TEM analysis was performed on the samples. Fig. 2 presents a cross-sectional TEM analysis of a stack containing the platinum (Pt) coating layer, SiO_2_, and Si of the as-deposited sample. The BF-TEM images in Fig. 2(a) enabled the determination of the SiO_2_ layer thickness, which was found to be 320 nm. Furthermore, the Pt layer deposited in the FIB can be seen effectively protecting the SiO_2_ layer. An HRTEM image of the SiO_2_/Si interface is presented in Fig. 2(b), and it reveals the presence of point defects in the Si region (marked by yellow dotted circles in Fig. 2(b)). The formation of point defects commonly occurs during the crystal growth process.^38,39^ The HRTEM image also reveals the single crystal of the Si substrates. The corresponding DF-STEM image of the stack region is presented in Fig. 2(c). Owing to its higher sensitivity to mass-thickness contrast, it showed the presence of voids in the SiO_2_ layer, which was not that clear in Fig. 2(a). These voids are formed due to dehydration resulting from the annealing process involved in the sol–gel process. This results in loss of water and alcohol contents, in addition to extracting voids/pockets. The range of void size was found to be around 15–20 nm. The elemental mapping of the stack, performed using STEM-EELS on a region, is shown in Fig. 2(d). The corresponding Red–Green–Blue (RGB) composite generated from Pt, O, and Si maps is presented in Fig. 2(e). The dark contrast in the SiO_2_ layer in Fig. 2(e) implies the presence of no elements at these locations, and thus, it reconfirms their identity as voids.

**Fig. 3:**
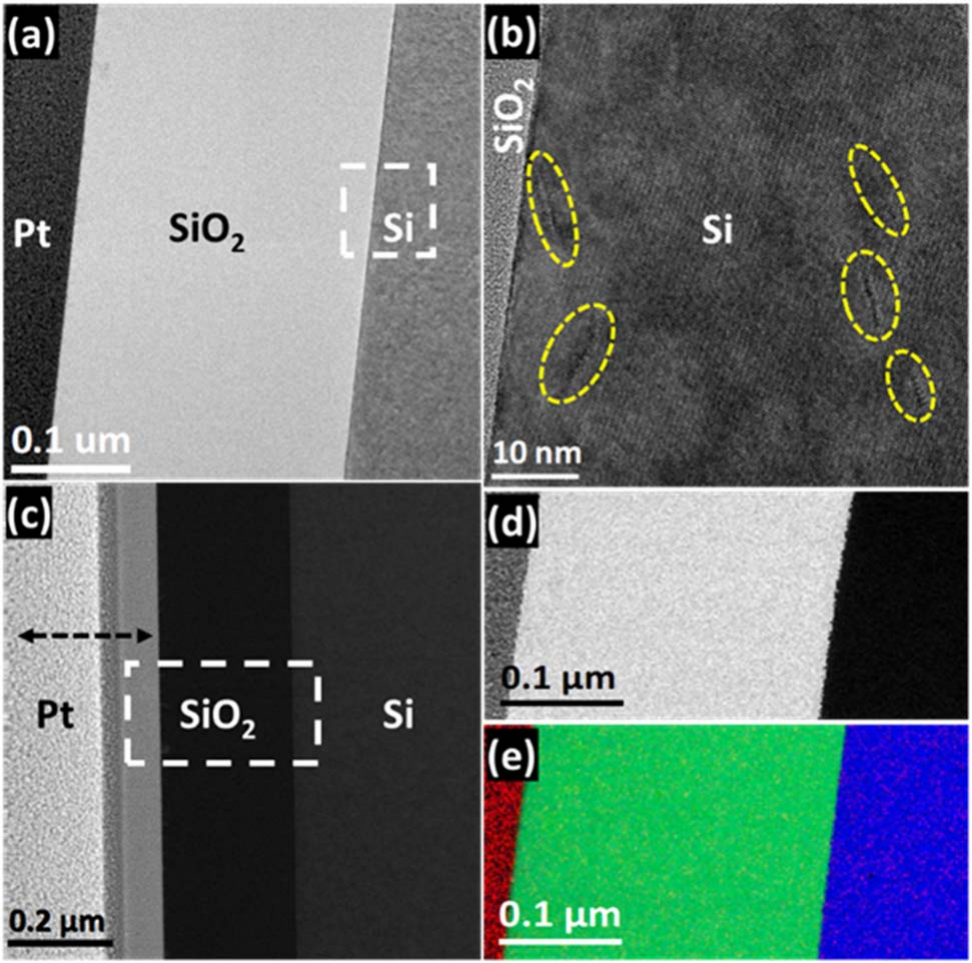
TEM analysis for the 900 °C annealed sample: (a) cross-sectional low-resolution image revealing SiO_2_ thickness around 250 nm and (b) HRTEM image showing line defects in the Si region. The presence of dislocations is marked by adding oval shape enclosed regions, (c) DF-STEM image exhibiting absence of voids in the SiO_2_ layer, (d) DF-STEM image of the area enclosed by a rectangular white-color box in (c) that was selected for the acquisition of STEM-EELS SI datasets, and (e) RGB composite generated with Pt (red), O (green) and Si (blue) elemental maps of the area represented by the DF-STEM image in (d).

The TEM analysis of the samples subjected to annealing at 900 °C is presented in Fig. 3. The BF-TEM image in Fig. 3(a) presents a low-resolution image of the stack. The depicted image reveals a higher quality of the stack and enables the measurement of the SiO_2_ layer thickness, which turned out to be approximately 250 nm. The apparent reduction in the thickness of the SiO_2_ layer reveals the evaporation of water that gets incorporated during the sol–gel synthesis method. In other words, the annealing of the samples leads to the removal of water molecules trapped in the SiO_2_ layer. It simultaneously makes the layer more compact, thus resulting in a more stable and higher-quality SiO_2_ layer. Consequently, no void formation occurs in the SiO_2_ layer, but its thickness reduces. The HRTEM image of the SiO_2_/Si interface is presented in Fig. 3(b), which shows the atomic structure of the stack at high resolution. The given image nicely demonstrates a single-crystal structure of the Si substrate. Moreover, the annealing of the point defect resulted in the formation of line defects or dislocations enclosed by yellow lines. The density of the dislocations was determined from the analysis of HRTEM images. A few orders of magnitude less than the density of point defects were found in the as-deposited samples *via* HRTEM analysis. Quantitatively, in this case, the density of point defects was approximately estimated as 10^20^ per cm^3^ and around 10^16^ per cm^3^ in the as-deposited and annealed samples, respectively. The DF-STEM analysis depicted in Fig. 3(c) provides visual evidence indicating the absence of voids in the SiO_2_ layer after annealing. Fig. 3(d) illustrates the elemental mapping of the stack conducted using STEM-EELS in a specific region. At the same time, Fig. 3(e) presents the resulting RGB composite generated from Pt, O, and Si maps. The changes observed in the structural and elemental properties of as-deposited and annealed samples surely affect the emergent properties of the stack. Dislocations at the SiO_2_/Si interface can exert mixed or even detrimental effects on the structural and electronic properties of the Si-substrate. On the one hand, dislocations can partially relieve accumulated strain by enabling localized lattice relaxation, which helps reduce large-scale mechanical stress. On the other hand, their presence often introduces undesirable consequences: they disrupt crystallographic order, generate localized strain fields, and can act as scattering centers or recombination sites for charge carriers. Such defects degrade carrier mobility, reduce device reliability, and may shift the band structure in unpredictable ways. In strained semiconductor systems, dislocations therefore represent a double-edged sword—while they can accommodate strain, their detrimental impact on electronic performance often outweighs the benefits of partial stress relief.

**Fig. 4:**
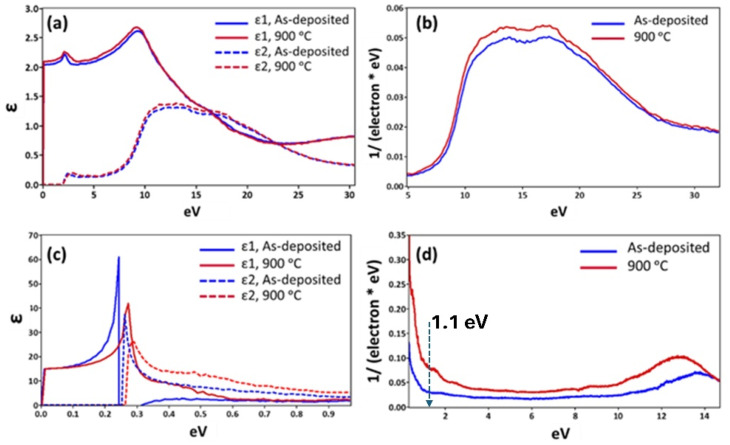
Dielectric properties of the SiO_2_ layers and Si substrate under as-deposited and annealed conditions were obtained by applying KKA to the STEM-EELS datasets. (a) Real (*ε*_1_) and imaginary (*ε*_2_) parts of the dielectric function *ε*, of SiO_2_ layers, (b) the corresponding JDOS plot, (c) *ε*_1_ and *ε*_2_ parts of *ε* of the Si substrate and (d) the corresponding JDOS plot.

The electronic properties of the SiO_2_ layer and Si substrate are the most critical factors that control the light emission efficiency observed with PL experiments. This is why the STEM-EELS technique, which also allows the investigation of the electronic properties of materials at the nanoscale, was applied to the samples presented in this study.^40,41^Fig. 4 shows the dielectric function and JDOS results of the SiO_2_ layer obtained by applying Kramers–Kronig analysis (KKA) to the ELF extracted from the EELS spectra of the SiO_2_ layer.^42,43^ It can be noticed from Fig. 4(a) that both the real and imaginary parts of the dielectric function slightly changed as a result of the annealing of the samples at 900 °C. This finding implies that the Si–O bonding in the SiO_2_ layer remains predominantly unchanged, and thus, only reorganization occurs within the layer. In this way, the STEM-EELS analysis of the stack revealed that optical parameters, such as the refractive index (*n*) at energies above the SiO_2_ bandgap, exhibit slight variations. Although annealing slightly enhances JDOS (see Fig. 4(b)), the enhancement within the energy range of 10 eV to 25 eV indicates that annealing alters the short-range order (SRO) in the SiO_2_ layer. This finding is expected since SiO_2_ annealing at high temperatures enhances its crystallinity, which becomes visible in the observed JDOS.^44^ Similarly, the dielectric properties and JDOS of the Si substrate in Fig. 4(c) and (d) show that these properties change significantly due to the annealing of the stacks at 900 °C. Specifically, the JDOS is expected to change nearly 2.7-fold at an energy of 1.1 eV, corresponding to a wavelength of 1160 nm, indicating a bandgap-emission enhancement in the Si substrate due to annealing. To confirm the 2.7× JDOS enhancement, the low-loss data acquisition and subsequent KKA analysis were repeated three times to demonstrate consistency and highlight the variation range. This will help convey both the robustness of the trends and the experimental uncertainties associated with the measurements. While the present study primarily addresses the optical impact of voids through Rayleigh scattering, it is important to note that such voids may also introduce trap states or act as non-radiative recombination centers at the Si/SiO_2_ interface. These potential electronic effects were not explored in this study and represent a limitation of the current work. Future investigations, such as carrier lifetime measurements or interface defect spectroscopy, could provide valuable insights into how void-induced recombination dynamics further influence the overall emission properties.

**Fig. 5:**
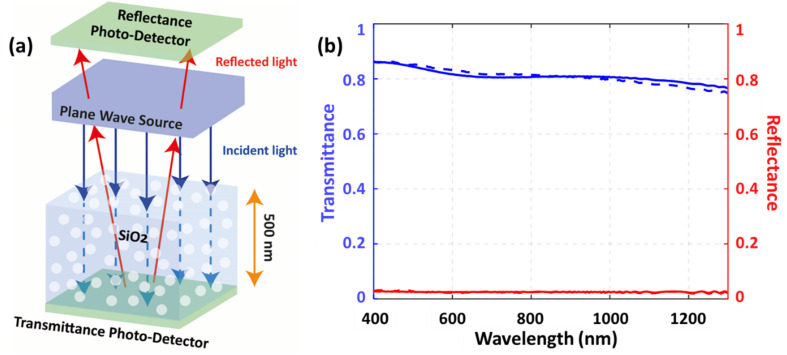
(a) Schematic diagram of the simulation model for the as-deposited sample and (b) simulated transmittance and reflectance for the SiO_2_ layer for as-deposited and annealed samples.

The subsequent discussion examines the impact of voids observed in the SiO_2_ layer of the as-deposited samples (Fig. 2(c)) on their optical properties. This was assessed through FDTD simulations, focusing on light transmittance and reflectance through the SiO_2_ layers of both samples. A schematic representation of the simulation model for the as-deposited sample is illustrated in Fig. 5(a), wherein randomly distributed voids were introduced into the SiO_2_ layer, and transmittance and reflectance detectors were placed beneath the SiO_2_ layer and above the plane wave source, respectively. A similar model was employed for the annealed samples, but without voids. The transmittance was computed using eqn (1):1
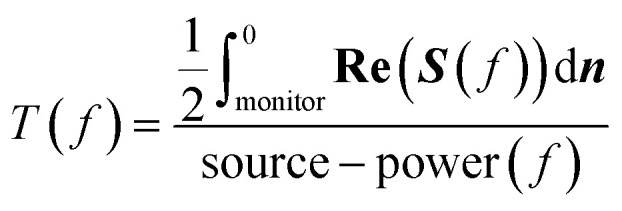
where ***S*** (*f*) is the Poynting vector at frequency (*f*), and ***n*** is the surface normal. Fig. 5(b) shows the simulated results of these optical properties as a function of the wavelength of light. The simulations were performed in the same wavelength range utilized in the PL experiments. Moreover, the simulations revealed that annealing results in a slight decrease in light transmittance through the SiO_2_ layer in the 400–600 nm wavelength range, whereas the trend reverses in the 700–1200 nm wavelength range. The trends observed in reflectance are complementary to those observed in transmittance because the sum of these two quantities is the total amount of light in the absence of absorption.^45^ The simulated results correlate nicely with PL data on the optical properties of the same samples (Fig. 1(a)). The observed variations in light transmittance and reflectance in the SiO_2_ layer can be attributed to Rayleigh scattering, a phenomenon rooted in the interaction of light with nanometer-size particles, which are generally much smaller than the wavelength of visible light.^46^ In the context provided, voids within the SiO_2_ layer act as scattering centers for incident light because their dimensions are orders of magnitude smaller than the wavelength of light. This scattering phenomenon alters the path of light passing through the material, decreasing light transmittance through silica. Therefore, Rayleigh scattering provides a fundamental understanding of how the presence of voids affects the optical properties of the sol–gel synthesized SiO_2_ layer, as evidenced by the changes in transmittance and reflectance detected in the simulated results.

**Fig. 6:**
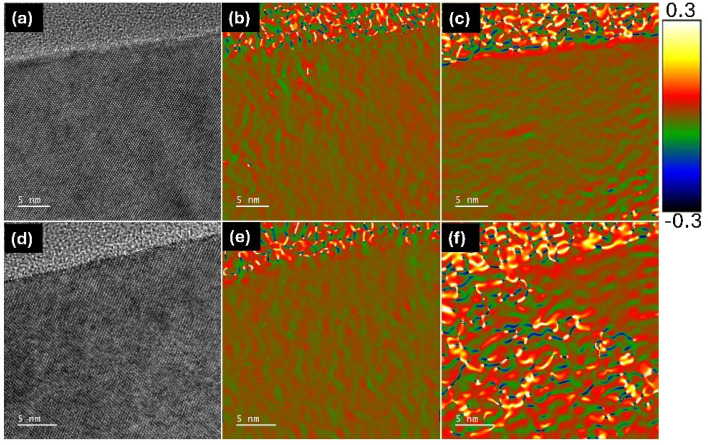
Strain mapping at the silicon interface: (a) HRTEM image of as-deposited samples, (b) strain map in as-deposited samples at the silicon interface in the 〈1̄10〉 direction, (c) strain map in as-deposited samples at the silicon interface in the 〈001〉 direction, (d) HRTEM image of 900 °C annealed samples, (e) strain map in 900 °C annealed samples at the silicon interface in the 〈1̄10〉 direction, and (f) strain map in 900 °C annealed samples at the silicon interface in the 〈001〉 direction.

Overall, the presented results demonstrate that the annealed samples exhibit higher-quality optical properties than the as-deposited samples, including the intriguing enhancement of bandgap emission. Specifically, under the same experimental conditions, the PL intensity of the annealed samples increases, which can be attributed to several reasons. First, point defects can act as centers for non-radiative recombination activities; therefore, lowering their density through annealing results in an increase in band-to-band recombination activities. It has also been shown that the point defects can become the centers for the creation of radiative recombination of charge carriers.^47^ However, point-defect-mediated radiation recombination does not significantly influence light-emitting properties in Cz-Si substrates.^48^ Nevertheless, the annealing of point defects results in the formation of dislocations that act as deep-level impurities to create sub-bandgap states, ultimately leading to an enhancement in the emission of light signals as observed in the PL data (see Fig. 1).^49^ Hence, it can be stated that annealing enhances the light-emission efficiency of Si by some fraction. PL study of the SiO_2_/Si stacks under thermal conditions demonstrates that strain build-up occurs at the SiO_2_/Si interface. This leads to a decrease in the Si bandgap value by as much as 0.15 eV per 1% change in the strain of the Si substrate, along with an accompanying increase in its refractive index.^50^ This reported finding suggests that applying strain may be a promising way to adjust silicon's bandgap and refractive index. The strain can be generated and released freely and quickly, and the refractive index change can reach 1% or even more. In addition, the shift of the Si bandgap from its intrinsic bandgap of 1.1 eV provides the possibility of making silicon-based photodetectors for a tunable wavelength range. The nature of strain buildup at the SiO_2_/Si interface due to annealing has been investigated by applying geometric phase analysis (GPA) to high-resolution TEM images of the Si interface.^51^ The GPA analysis presented in Fig. 6 provides a powerful way for extracting local lattice distortions directly from high-resolution images by analyzing the phase of the Fourier components associated with specific lattice fringes. By selecting reciprocal lattice vectors corresponding to particular crystallographic directions, *i.e.*, the 〈1̄10〉 direction in Fig. 6(b) and (d) and the 〈001〉 direction in Fig. 6(c) and (f), allowed determining the strain changes at the silicon interface as a result of its 900 °C annealing. For the as-deposited samples, the generated maps show a uniform strain field distribution (<±0.1%) both in the along and across directions of the SiO_2_/Si interface, *i.e.*, in the along (〈1̄10〉) and perpendicular (〈001〉) directions, respectively. Nevertheless, a small region at the SiO_2_/Si boundary showed fluctuations in the 〈001〉 direction, reflecting the presence of built-in mismatch strain due to lattice discontinuity at the interface. For the sample annealed at 900 °C (Fig. 6(d–f)), the HRTEM image (d) shows improved lattice ordering in the silicon near the interface. However, the GPA-derived strain maps (e and f) reveal a much stronger and more heterogeneous strain field (>±2.0%). Compared to the as-deposited case, the strain values fluctuate significantly, reaching both tensile (positive) and compressive (negative) regimes. This enhanced strain contrast arises from thermal processing, which induces atomic rearrangements, relaxation, and local defect formation at the SiO_2_/Si boundary. Such variations are evident in the 〈001〉 direction, Fig. 6(f), which reflects out-of-plane distortions. Therefore, it can be stated that the annealing process of the sol–gel-based silica and Si stacks in this study perturbs the Si bandgap randomly. Overall, the GPA analysis demonstrates that annealing not only modifies the structural coherence at the interface but also amplifies local strain heterogeneities. These strain fields are critical in determining the electronic and optical properties of the Si substrate, as they can alter the band structure, carrier mobility, and defect states. The observed random perturbation in the strain at the Si interface likely causes a shift in its electronic bandgap, which can result in the confinement of electron–hole pairs by binding them in a way that appears to decrease the three-particle interaction into two-particle interactions of e–h and phonons.^3,52,53^

The ∼2% enhancement in the transmittance of light at the wavelength of 1160 nm through a 900 °C annealed SiO_2_ layer indicates that the SiO_2_ layer also contributes marginally to the collective enhancement in the signal observed in the PL experiments. It also means that the overall four-fold enhancement of PL intensity in the present study is correlated with the changes in the SiO_2_ layer due to annealing at a temperature of 900 °C. The presence of voids in the SiO_2_ layer turns it into a sort of porous silica, and the light transmittance of porous silica materials decreases with increasing thickness. It has been shown *via* simulations that the spherical voids in silica result in a decrease in transmittance quadratically with increasing propagation depth, generally.^54^ In this way, morphological features such as voids affect light transmittance in various ways, including the refractive index of air in the voids, the size of the voids, and the wavelength of light. In addition, the FDTD calculations presented herein show that the transmittance of light decreases by nearly 30% through the SiO_2_ layer with voids of an average size of 15 nm compared to the case with no voids. Therefore, the rest of the enhancement, which is about 2.8-fold, in the observed PL intensity is due to the changes taking place in bandgap emission properties of the Si substrate, specifically a 2.7-fold enhancement in the Si JDOS at the bandgap energy (see Fig. 4(d)).

Ultimately, our study showcases an effective strategy for fabricating high-quality devices using cost-effective methods such as the sol–gel technique. The sol–gel method provides a practical approach to producing high-quality SiO_2_ layers on silicon substrates. Upon annealing, the resulting stacked structure exhibits exceptional physical properties, as thermal treatment facilitates the removal of residual solvents. This underscores the potential of inexpensive techniques in advancing the development of next-generation electronic devices. The demonstrated sol–gel-derived SiO_2_/Si stacks are not only effective for enhancing silicon photoluminescence but are also inherently compatible with conventional silicon processing. This CMOS compatibility makes the approach attractive for future integration into silicon photonic platforms, where on-chip light sources remain a critical challenge. Recent advances in strained silicon emitters and defect-center-based light sources have underscored the importance of CMOS-integrable solutions for scalable photonic systems.^55,56^ Moreover, optical interconnects, which require efficient, low-cost emitters directly on silicon, could particularly benefit from sol–gel-processed SiO_2_/Si structures due to their low-temperature fabrication and compatibility with wafer-scale integration.^57,58^ Thus, beyond their immediate photonic performance, the methods reported here represent a practical step toward bridging fundamental silicon emission challenges with the requirements of next-generation CMOS-compatible optical interconnect technologies.

## 4 Conclusions

The annealing of sol–gel prepared SiO_2_/Si stacks was found to play a crucial role in determining the light emission properties of the Si bandgap. We have demonstrated that sol–gel-derived SiO_2_ coatings significantly modify the structural and optical properties of the Si/SiO_2_ interface. GPA strain analysis of HRTEM images revealed that annealing at 900 °C induces local strain fluctuations of up to ±2%, compared to the relatively uniform strain (<±0.1%) in the as-deposited samples. This enhanced strain field correlates with a nearly fourfold increase in photoluminescence (PL) intensity from the annealed Si/SiO_2_ structures, indicating strain-mediated modifications to the band structure. Simulated optical measurements confirmed that the sol–gel SiO_2_ layer remains highly transparent, with an average transmittance exceeding 75% and reflectance below 5% across the 400–1300 nm range, thereby minimizing parasitic optical losses. Moreover, electronic calculations showed a 2.7× increase in the joint density of states (JDOS) at relevant transition energies after annealing, consistent with the observed PL enhancement. These results provide direct evidence that strain engineering at the nanoscale, induced through sol–gel processing and controlled annealing, can substantially enhance silicon emission without compromising optical transparency, offering a promising pathway for silicon-based photonic applications. Furthermore, given its simplicity and compatibility with existing silicon processing, this methodology could be readily adapted to CMOS photonics, enabling efficient on-chip emitters and advancing future optical interconnect technologies.

## Conflicts of interest

The authors declare that there is no conflict of interest.

## Data Availability

Data for this article is available under the title NA-COM-05-2025-000472_R1, which includes the entire data in raw formats at the Science Data Bank with URL: https://www.scidb.cn/en/detail?dataSetId=65058614eba94662acb1340325c174ca and DOI address: 10.57760/sciencedb.29892.
